# Treating COVID-19 Positive Cancer Patients With Radiation Therapy: A Case Report From Epicenter of the Pandemic

**DOI:** 10.7759/cureus.11967

**Published:** 2020-12-08

**Authors:** Bhupesh Parashar, Lucille Lee, Chika Madu, Ashwatha Narayana, Louis Potters

**Affiliations:** 1 Radiation Oncology, Zucker School of Medicine at Hofstra/Northwell, Hempstead, USA

**Keywords:** covid-19, radiation, cancer, pandemic, positive

## Abstract

The coronavirus disease 2019 (SARS-Cov-2 or COVID-19) pandemic has resulted in unprecedented clinical challenges across the globe. Outcomes of patients with this infection are likely dependent on underlying comorbidities that predict worse outcome in older patients. However, it is unknown whether COVID-19 infected cancer patients receiving radiation therapy (RT) have any different outcome than non-infected patients. We present the first series from our center of COVID-19 infected patients who received RT for malignancy, their outcome, and toxicities.

## Introduction

The coronavirus disease 2019 (COVID-19) pandemic has resulted in unprecedented challenges globally, including social, healthcare, economic, and cultural issues. It is caused by a virus called severe acute respiratory coronavirus 2 (SARS-COV-2) [1]. The virus has been sequenced, and many groups worldwide are involved in studying the virus, the development of detection kits, the development of antibody tests, and vaccine development [2]. Regarding cancer patients who get infected with the COVID-19 virus, it is not clear whether the virus has any impact on cancer progression, tolerance to chemotherapy or radiation therapy (RT), and overall prognosis [3]. The pre-print of the article is available on research scholar (https://www.researchsquare.com/article/rs-35237/v1).

In this study, we document our experience from the epicenter of the pandemic in treating four COVID-19 positive patients with RT. Data were collected through our ONCORA data collection system that is integrated with our electronic medical records.

## Case presentation

Case 1 

A 76-year-old female was newly diagnosed with FIGO (International Federation of Gynecology and Obstetrics) stage IIA cervical clear cell adenocarcinoma. Work-up, including a PET-CT (positron emission tomography-computed tomography), showed a large cervical mass (10 cm) with no distant disease. The patient was recommended to undergo chemoradiation therapy (CRT). Initial RT consult was in February 2020 for vaginal bleeding and management of cervical cancer. She was planned for a total external beam radiation therapy (EBRT) dose of 4,500 cGy (180 cGy/fraction) followed by brachytherapy. On RT treatment (12 fractions out of 25 total fractions), the patient started showing signs of labored breathing, and oxygen saturation was 88% on 2 L oxygen while lying flat. When brought back to a high Fowler's position, oxygen saturation increased to 96%. Axillary temperature was 101.6°F. RT was withheld, and COVID-19 antigen test was performed, which was positive. She was admitted to the hospital and she subsequently developed abdominal distension and possible perforation. She then developed pulmonary symptoms and died subsequently. The cause of perforation was not confirmed, and the patient passed away without completing her RT.

Case 2 

A 70-year-old female with T3N2bM0 stage IIIB was diagnosed with unresectable squamous cell carcinoma of the vulva. In December 2019, the patient was noted to have a vulvar bump that was becoming increasingly painful. Work-up including a CT of the chest/abdomen/pelvis and PET-CT showed mildly enlarged left common iliac lymph node, 1.0 cm in short axis and a markedly enlarged left inguinal 4-cm lymph node in addition to the primary lesion. The treatment team recommended CRT. 5-Fluorouracil/mitomycin C was recommended since the patient was not deemed a good candidate for cisplatin. The patient was planned for EBRT with intensity-modulated radiation therapy to a dose painting of 6,400 cGy (to gross disease) in 2 Gy/fraction plus pelvic and inguinal nodes. The patient started CRT in mid-March 2020. Treatment showed good response. Treatment break was given at the end of March 2020 for five days for desquamation of skin at the treatment site. The patient returned to treatment, but in the second week of April 2020, she was diagnosed with COVID-19 by a polymerase chain reaction (PCR) test after developing shortness of breath. The patient's oxygen saturation decreased overnight but improved to 92% on 2 L oxygen. The patient's inpatient note was recorded as 'Patient admitted for hypoxemia secondary to COVID and supratherapeutic INR due to hypercoagulative state secondary to COVID and using Coumadin®. The patient was prescribed Plaquenil®, and her symptoms improved.

The patient’s RT treatment was restarted, and she completed 56 Gy in 28 Gy per fractions. The patient's treatment was interrupted due to poor nursing care at the facility (possibly due to her COVID-19 diagnosis), and she decided to stop the treatment. On the last examination before she stopped treatment, she had a nearly complete clinical response.

Case 3

The patient was a 65-year-old female with FIGO stage IV (TNM stage: T3 N2 M1) endometrial carcinoma with sarcomatoid features. She presented with post-menopausal bleeding and was hospitalized from April 2020 to early May 2020. Work-up including an endometrial biopsy showed superficial fragments of high-grade malignant neoplasm with sarcomatous features. CT of the abdomen and pelvis showed intrauterine 11.6-cm partially necrotic mass with extrauterine local invasion, suspicious for primary endometrial neoplasm. There were extensive peritoneal tumor implants with a dominant 8-cm pelvic implant. In addition, there were multiple bilateral solid pulmonary nodules, suspicious for pulmonary metastases plus retroperitoneal metastatic lymphadenopathy. Given her metastatic disease, palliative chemotherapy using carboplatin/Taxol® was planned, and the radiation team was consulted for local treatment for continuing vaginal bleeding. The patient was treated to the pelvis/involved uterus/cervix using a 3D conformal technique (2,000 cGy in five fractions). During her RT course, she presented with cough, and a chest X-ray revealed low lung volumes plus reticular densities in the left lung base. The patient was diagnosed with COVID-19 by PCR. She did not require hospitalization. Her vaginal bleeding improved toward the end of her treatment course. The course of RT was completed without any complications.

Case 4

The patient was a 76-year-old male with a history of Gleason 8 prostate cancer initially diagnosed in 2014. He received pelvic RT for his localized disease. He was then diagnosed with metastatic disease in 2015 and was on androgen deprivation therapy (ADT). The patient’s past medical history was significant for diabetes mellitus, deep venous thrombosis, and hypercalcemia secondary to osseous metastasis. The patient had received a second course of RT to the left iliac bone in January 2020 for pain palliation. In early May 2020, he tested positive for COVID by PCR during hospital admission for back pain. He was given palliative RT to T6-T10 vertebral body to 8 Gy/fraction during this admission. The patient tolerated the RT treatment well and responded with improved pain control in the treated region. There were no adverse effects of RT.

The four cases are summarized in Table 1.

**Table 1:** COVID-19 positive patients receiving RT RT, radiation therapy

Case No.	Primary Site	Site of RT	Total Dose/Dose per Fraction	Intent	Alive/Died	Comments
1	Cervix	Cervix plus nodes	21.6 Gy/1.8 Gy/fraction; planned 45 Gy	Curative	Died	Perforation detected; died of pulmonary symptoms secondary to COVID-19
2	Vulvar cancer	Vulva plus nodes	56 Gy/2 Gy/fraction; planned 64 Gy	Curative	Alive	Stopped treatment at 56 Gy due to poor nursing home care; clinical complete response
3	Endometrial	Uterus	20 Gy/4 Gy/fraction	Palliative	Alive	Doing well, bleeding stopped
4	Prostate	Thoracic spine	8 Gy/8 Gy x 1 fraction	Palliative	Alive	Doing well; planned for hospice

## Discussion

Our case series of four patients who were COVID-19 positive and received RT had outcomes that do not seem to be affected by this infection. There were no acute unexpected adverse effects from RT noted in this group of patients versus those previously treated before this pandemic, although one patient had bowel perforation that has recently been reported to have some association with COVID-19 infection [4]. Our report is one of the first reported series of COVID-19 infected cancer patients treated with RT. Table 2 outlines the guidelines to manage COVID-19 positive patients in the radiation department.

**Table 2:** Guidelines to manage COVID-19 positive patients in the radiation department CDC, Centers for Disease Control and Prevention; PPE, personal protective equipment; PUI, persons under investigation

Guidelines
For asymptomatic patients:
Follow standard "Ask and Mask" guidelines at check-in.
Patients will be offered a mask anyway, even if asymptomatic, while in the department. We understand that many cancer clinics are mandating masks for all patients given the asymptomatic spread of coronavirus.
For known positive patients or patients awaiting test results (PUI):
Must treat the last patient of the day.
Ask the patient to text department upon arrival to the parking lot and the patient will be given a surgical mask and gloves upon arriving for treatment. CDC requires hand hygiene but gloves are an option.
Staff members caring for patients will meet them outside of the building daily wearing full PPE: N95 respirator/surgical mask, isolation gown, and gloves, eye shields.
A staff member will escort the patient through the back of the department and enter through the exit door on the side of the radiation treatment room.
If the patient needs to change into a gown for treatment, the patient will change inside of the treatment console to avoid exposure to other areas.
Post-treatment, the patient will be escorted out of the department wearing a surgical mask.
A staff member to remove all PPE post-treatment outside of the department and place it in a waste container with a lid to be discarded and perform hand hygiene.
Cleaning of room after the patient leaves:
Room door must remain closed, and the room remains unoccupied for one hour before entering. This duration may vary based on clinical situation (e.g., patient coughs or there is additional risk of exposure).
All horizontal surfaces to be cleaned using grey top PDI wipes (PDI Inc., Woodcliff Lake, NJ, USA) by environmental services. Any surface that touched the patient, immobilization devices, and other radiotherapy accessories can be disinfected with disposable disinfecting wipes or 75% ethanol.
Caring for more than one positive patient:
Cleaning instructions as outlined above must be followed in between two positive patients.
Room to remain unoccupied with the door closed for one hour.
All horizontal surfaces to be cleaned as per protocol above.

Our case series is unique because we have treated COVID-19 infected patients with radiation for various malignancies (cancers of the cervix, endometrium, vulvar, and prostate). Given the novelty of the virus and its potential impact on the outcomes of cancer patients, our case report will add to the literature and knowledge about this deadly infection. We had a few concerns about treating COVID-19 positive patients with radiation: (1) does COVID infection make radiation effect on cancer any different for infected versus non-infected patients? and (2) how can we safely deliver radiation to patients who are infected with this highly contagious virus?

The cervix cancer patient (case 1) died of pulmonary complications of COVID and could not complete the radiation treatment. The patient did develop bowel perforation, although it is not clear if it was secondary to COVID. No autopsy was performed, and there was no documentation of clinical decision to suggest a Covid-19 association with the perforation. Since this is a case report with a single patient showing this complication, we will have to wait for a larger series of patients to determine if there is an association between the two.

The next patient (case 2) with vulvar cancer did not develop any complications from radiation treatment, although could not complete her radiation course due to poor personal care at the nursing home. The patient decided to stop her treatment with a few remaining radiation fractions, although she had a complete response to RT. This is not an uncommon clinical challenge in nursing home patients even prior to the COVID pandemic, although one can hypothesize if the lack of personal care was secondary to fear among the nursing home staff about catching the infection from the infected patient. There was no evidence found for this “fear among staff” explanation.

The endometrial and prostate cancer patients (cases 3 and 4, respectively) were treated palliatively and responded well to the treatment with improvement in their symptoms.

We followed the departmental guidelines for staff safety during radiation delivery to COVID-19 positive cancer patients, as outlined in Table 2. Once the policies were implemented, we did not see any staff catching the virus from any infected patients. We believe that the recommendations outlined in Table 2 are quite robust in keeping the staff safe while treating COVID-19 infected cancer patients and can be used across the country in radiation departments.

Select relevant studies in the following discuss the management of cancer patients infected with COVID-19 and treated with systemic treatments or surgery. A report published in February from China showed a prevalence of 1% cancer in COVID-19 infected patients versus 0.29% in the general population. The most frequent site of malignancy was the lung, and patients were likely to be older and smokers [1]. This group of cancer patients was more likely to experience severe events from treatment. However, with only 18 patients identified in the study, the conclusions of the study are unreliable [5].

Regarding managing cancer patients who develop COVID-19 infection, a modeling study [6] using a Markov model estimated ICU requirements for cancer patients to be 12.7 days, the median time to mortality to be 16.3 days, and the median time to severe events to be 8.1 days. A French registry study [7] to estimate the incidence of COVID-19 infections in breast cancer (BC) patients showed that 76 actively treated BC patients out of 15,600 BC consultations before lockdown. Of the 59 COVID-19 patients (76 BC patients), 17% (10/59) were >70 year of age and 47% were hospitalized. Four patients were transferred to ICU, 76% were considered cured or recovering, and 6.7% of patients died. A case report of COVID-19 positive [8] a patient who received adjuvant immunotherapy after complete resection of stage IV melanoma had mild symptoms that resolved spontaneously in three days and the patient remains tumor-free. In a study [9] of patients with CML (chronic myeloid leukemia) receiving TKI-therapy (tyrosine kinase inhibitor therapy) from Hubei Province, China, the COVID-19 prevalence of 0.9%, which was nine-fold higher than 0.1% reported in the general population.

In a case series [10] of 11 patients who underwent thoracic surgery and were consequently diagnosed with COVID-19, there was a median of 13 days from surgery to COVID-19 infection (range: 1-31 days), 35 days from surgery to death (range: 5-42 days; n = 3), and 50 days from surgery to discharge upon recovery (range: 38-72 days; n = 8). Case fatality (27.3%) was high and was significantly associated with resected lung segments (≥5), the severity of postoperative hypoproteinemia or hypoalbuminemia, and the peak value of lactate dehydrogenase.

In a case series of patients [11] infected with COVID-19 from New York, 27/4,515 (0.6%) were diagnosed with BC. The majority had stage I to III BC and 19% had metastatic disease. In the six months before COVID-19, 59% received chemotherapy, 44% received hormone therapy, 22% received HER2-directed therapy, 4% received checkpoint inhibitor, 22% underwent breast surgery, and 7% received RT. Treatment disruptions occurred in 74% of patients due to COVID-19. With a median follow-up from COVID diagnosis of 26 days (range: 1-38 days), all patients were alive, except for an 87-year-old male with multiple comorbidities who received taxane-based chemotherapy for stage II BC seven days before symptoms.

## Conclusions

Our study is the first reported case series of cancer patients who were COVID-19 positive and received RT. Our limited experience suggests no direct added toxicity during COVID-19 infection, although long-term data are pending. In the future, prospective studies using RT in COVID-19 patients will be ideal.
